# Perception of Risk, Self-Efficacy and Social Trust during the Diffusion of Covid-19 in Italy †

**DOI:** 10.3390/ijerph18073427

**Published:** 2021-03-25

**Authors:** Pierluigi Diotaiuti, Giuseppe Valente, Stefania Mancone, Lavinia Falese, Fernando Bellizzi, Daniela Anastasi, Elisa Langiano, Fábio Hech Dominski, Alexandro Andrade

**Affiliations:** 1 Department of Human Sciences, Society and Health, University of Cassino and Southern Lazio, 03043 Cassino, Italy; giuseppe.valente@unicas.it (G.V.); s.mancone@unicas.it (S.M.); l.falese@unicas.it (L.F.); fernando.bellizzi@unicas.it (F.B.); d.anastasi@unicas.it (D.A.); langiano@unicas.it (E.L.); 2 Department of Physical Education, Univille University, Joinville 89219-710, Brazil; fabiohdominski@hotmail.com; 3 Health and Sports Science Center, Department of Physical Education, CEFID, Santa Catarina State University, Florianópolis 88035-901, Brazil; alexandro.andrade.phd@gmail.com

**Keywords:** Covid-19, institutional trust, interpersonal trust, locus of control, risk perception, self-efficacy, self-restraint behaviour, social distancing, traits

## Abstract

The Coronavirus pandemic has affected the lives of people all over the world. The perception of risk and people’s consequent behaviour during a pandemic are very complex and are affected by multiple cultural and psychological factors. The aim of this study was to investigate the change in risk perception, perceived self-efficacy and the perceived trust in the behaviour of others, the decisions of health authorities and government provisions, as well as the variation of self-restraint behaviours during the spread of the Covid-19 epidemic in Italy. We used a convenience sample of 707 university students (M_age_ = 22.99; SD = 4.01) from a central area of Italy. Participants freely joined the research by answering an online questionnaire between February and March 2020. Three time intervals defined by the progressive containment measures implemented by the Italian Government were considered. Main outcome measures were the Generalized Self-Efficacy Scale, the Risk Perception Index, the Index of Self-restraint Behaviours, and Institutional and Interpersonal Trust Measures. Results confirmed that significant changes in the time progression have occurred in the perception of risk, in the perception of individual self-efficacy, in the value attributed to social responsibility, in interpersonal trust and in trust in health authorities. The study also identified the participants’ personality traits and locus of control as predictors (positive and negative) of perceived self-efficacy and tested a mediation model of trust on the effect of risk perception on self-restraint intentions.

## 1. Introduction

The Covid-19 coronavirus pandemic has now affected the lives of people all over the world. The perception of risk and people’s consequent behavior during a pandemic are very complex and are affected by multiple cultural and psychological factors [1,2]. Individuals’ perception of risk refers to a subjective cognitive evaluation process of the characteristics, severity and possible consequences and management of hazards they might be exposed to [3]. Perception of risk is influenced by social and cultural factors, experiences, beliefs, knowledge, and attitudes. Perception of risk was found inversely associated with self-efficacy in a study on a previous epidemic, the avian influenza: the higher the self-efficacy, the lower the perception of risk [4]. Perception of risk and self-efficacy in turn influence the engagement in precautionary actions during outbreaks [3,5]. Moreover, previous studies showed that self-efficacy is an important element in promoting health-related intentions and behaviours [6,7]. As a pandemic spreads, people can take a number of precautionary measures to avoid infection, including social withdrawal [8] and/or immunization, when a vaccine is available. They often judge personal risk based on their impressions of the overall prevalence and severity of the disease [9,10]. A preliminary study on risk perception related to Covid-19 in Germany showed that older people and the male gender have a lower risk perception than younger people and women [2]. Over time, the perception of the lethality of the event may be diminished by the awareness of an inadequate or disorganized initial response.

Personality factors can also play a key role in predicting psychological health and resilience during a crisis. For example, regardless of socio-economic level, the degree to which individuals believe they can control events and outcomes in their own lives was found to be related to the process of coping with potentially threatening situations [11]. People who feel they have control of the situation, assessed with internal locus of control scores, better cope with all crises and disasters because they consider themselves masters of their own lives and destiny [11]. On the contrary, those who are classified with an external locus of control consider themselves victims of fate, with little perceived self-efficacy in influencing many events and outcomes of life [12]. Furthermore, studies on pro-social vs. selfish behavior have shown that when there is no certainty that a selfish action can lead to a potentially negative result for others, individuals are much more likely to act selfishly than when there is certainty [13]. Such reductions in pro-sociality may occur because uncertainty allows people to adopt selfish narratives that induce them to act selfishly while maintaining a positive self-image [14,15].

Consistent with this idea, when the results of decisions are uncertain, people optimistically underestimate the possibility that selfish behaviour causes negative results for others, making selfish behaviour more appropriate for themselves [16]. Perceptions of social norms (i.e., shared beliefs about what people should do in a given situation) reflect these results: selfish behaviour, when results are uncertain, not only seems appropriate to oneself, but also to others [17].

In order to better understand the development of the pandemic it could also be very useful to investigate the value attributed to social responsibility, interpersonal and institutional trust and confidence in health authorities. A change in the rate of confidence and trust can be related to different time periods throughout the development of the pandemic and the corresponding adopted restrictive measures [18].

The Coronavirus disease was identified in December 2019 in Wuhan, China and spread all over the world at the beginning of 2020. Italy (especially the northern areas) was one of the first countries in Europe to be contaminated and this type of health emergency was a completely new experience to tackle. The Government implemented progressive containment measures as the numbers of cases increased. At the time of our data collection, these measures can be categorized in three time intervals.

The first aim of this study was therefore to investigate the change in risk perception, perceived self-efficacy and the perceived trust in the behaviour of others, the decisions of health authorities and government provisions, as well as the variation of self-restraint behaviours during the spread of the Covid-19 pandemic in university students in Italy; whereas a second objective was to identify predictors of perceived self-efficacy and behavioural self-restraint intentions.

### Hypotheses

It was hypothesized that the progression of the implementation of national governmental restrictive measures during the first month of the spread of the Covid-19 pandemic in Italy would gradually increase the perception of the risk of being infected and gradually decrease the perception of individual self-efficacy, the value attributed to social responsibility, the interpersonal social trust and the trust in health authorities.

According to the role of hazard proximity and personal experience on risk perception described in previous scientific papers, we expected the perception of risk to be lower in the first ten days of the data collection since the restrictive measures concerned mostly northern Italy and our sample was from central Italy. A longer distance from the risk made it less probable for the respondents to have known someone infected or experience the disease themselves [19].

We then expected to see an increase in risk perception as soon as the restrictions were applied to the entire country, with the establishment of the closure of schools and universities and the limitation of social contact, even though we believed that some people would still claim that school and university closures are not an effective measure against the infection [20,21]. We also expected that individuals who feel they can control the outcome of a hazard would be less likely to be afraid of the disease [22].

Finally, in addition to the direct effect of risk perception on intentions to limit one’s behavior [3,5,6,23], we expected a mediating role of the (interpersonal and institutional) trust variable on this relationship [24,25,26,27,28]. Thus, we expected that an increase in risk perception would correspond to an increase in precautionary behavior intentions in the participants, at the same time as an increase in the trust component (interpersonal and institutional). The mediation hypothesis also involved the expectation of an influence of the trust component on Self-Restraint intentions.

## 2. Materials and Methods

### 2.1. Data Collection

We collected data corresponding to one month, from 25 February 2020 until 25 March 2020. For the data analysis, we divided this time period into three intervals according to the implementation of the containment measures adopted.

The first period runs from 25 February to 3 March 2020 and coincides with the initial Covid-19 contagion spread mainly in Northern Italy (Lombardy, Veneto, Piedmont and Emilia Romagna).

The second period runs from 4 March to 8 March 2020 and applies to measures valid for the entire national territory, such as the suspension of teaching activities in all schools and universities for 15 days. In northern Italy there was a ban on crowded events and an observance of safety distances, as well as restrictions on access to health facilities and prisons by relatives and visitors.

The third and final period taken into consideration in the study begins on 9 March 2020, the date of the implementation of the strongest restrictions in Northern Italy (total closure of Lombardy and fourteen provinces), and the extension to the rest of Italy of the measures to close pubs, cinemas, discos, events and sports competitions. Starting from 13 March, any travel not considered essential (except for the purchase of food and medicine, health and work reasons, and if the activity is considered essential and authorized) was prohibited throughout the country. The date of 25 March marks the closure of the data collection for this study.

### 2.2. Tools

Participants had to fill in an online questionnaire composed of the following sections:

(1) Socio-demographic information: gender; age; (2) area of residence (city > 50,000 inhabitants, town < 50,000 inhabitants), small town < 5000 inhabitants); (3) presence of chronic diseases (diabetes, asthma, disability, etc.); (4) means of transport mainly used (public, private); (5) average duration of daily trips using means of transport (less than one hour, between one and two hours, more than two hours); (6) Risk Perception Index (RPI): seven Likert 1–5 scale items, i.e., “How do you think the Coronavirus is likely to spread epidemically to the territory in which you live?”; “How much do you fear that you and/or your family may become infected?”; “To what extent have you changed your daily life in the last few weeks because of the possibility of Coronavirus infection?”; “Do you think that individual protection measures are a duty to the community?”; “How much do you think the risk of being infected during sports activities has increased?”; “To what extent have you changed your attitude towards people with flu symptoms since the Coronavirus spread?”; “How intensively do you try to avoid being close to people with flu symptoms such as coughing and sneezing?”. Altogether the seven items were grouped into a unique risk perception indicator. The index was subjected to a PCA exploratory verification and showed a bi-factorial structure, Determinant 0.269, KMO 0.820, Rotated Component Matrix Oblimin, Test Bartlett Sphericity Sig. 0.000 (Cronbach’s alpha = 0.71); (7) Institutional and Interpersonal Trust Measures (IIT): six Likert 1–5 scale items, i.e., “To what extent do you trust that people are adopting appropriate behavior to prevent the spread of the Coronavirus?”; “To what extent do you trust the efficiency of health authorities in managing the emergency?”; “To what extent do you trust the current measures adopted by the government?” PCA exploratory verification showed a mono-factorial structure, Determinant 0.124, KMO 0.809, Rotated Component Matrix Oblimin, Test Bartlett Sphericity Sig. 0.000 (Cronbach’s alpha = 0.72); (8) Index of Self-Restraint Behaviours (ISRB): seven Likert 1–5 scale items, i.e., If there were an outbreak of Coronavirus in the area in which you live, to what extent would you implement the following behavior: a. Avoid public transport; b. Avoid public venues; c. Avoid medical practices; d. Stay at home; e. Limit purchases; f. Avoid direct contacts; g. Be absent from work/university. PCA exploratory verification showed a monofactorial structure, Determinant 0.006, KMO 0.913, Rotated Component Matrix Oblimin, Test Bartlett Sphericity Sig. 0.000 (Cronbach’s alpha = 0.92); (9) the Generalized Self-Efficacy Scale (GSES); ten Likert 1–4 scale items validated in Italy by Sibilia et al. [29]. The scale assesses the general sense of perceived self-efficacy in order to predict coping with daily hassles as well as adaptation after experiencing all kinds of stressful life events (Cronbach’s alpha = 0.87); (10) the Locus of Control of Behavior (LCB) [30,31], composed of 17 items with a rating scale from 0 to 5; it measures the “place of control” (internal/external) of one’s own behavior that the subject generally uses in various situations. The scale has shown good reliability with Cronbach’s Alpha values of 0.70 for the internal locus and 0.73 for the external locus; (11) the *ITAPI-S*, short version of Italia Personality Inventory [32]. The instrument is composed of 28 items with Likert scale ranging from 1 (strongly disagree) to 4 intervals (strongly agree) and measures seven factors-traits, which are: dynamism (initiative, curiosity, vivacity); vulnerability (discomfort, fear, suffering); empathy (solidarity, sociability, sensitivity); conscientiousness (perseverance, precision, rationality); imagination (creativity, feeling, fantasy); defensiveness (distrust, rigidity, materiality); introversion (introspection, self-sufficiency, isolation). The scales showed good reliability with Cronbach’s Alpha values between 0.69 and 0.72.

### 2.3. Participants and Questionnaire Administration Procedures

Students of the local university were involved in a representative proportion (at least 30%) of the three major regions of origin (Lazio, Campania, Molise). For the purposes of the study, the university administration granted access to the database of email contacts of enrolled students; the database also contained information on the residence of these students. Taking into account the regional distribution indicated above, and the area of study (representing at least 20% of the five areas of study present in the university: economics, law, humanities, engineering and health), three thousand emails were sent out (February 25), extracting them from a list of approximately 9000 contacts. Participants therefore received an email inviting them to freely join the research by answering an online questionnaire. They were assured anonymity and the use of data in aggregate form for research purposes only. It was specified that they would not receive remuneration for their participation and if they had any doubts or problems they could contact the study representative directly. The average completion time was about 15 min. Tools administration took place upon the release and signing of the form for an informed consent of participation in accordance with the Declaration of Helsinki. The study received approval from the Institutional Review Board of the University of Cassino. On March 25 (which marked the conclusion of the data collection) there was a total of 707 participants (58.3% females) aged between 18 and 36 years (M = 22.99; SD = 4.01).

A total of 6.2% of the participants resided in cities (>50,000 inh), 35.2% in towns (between 5000 and 50,000 inha), 58.6% in small towns (<5000 inh). As regards general health information, 7.1% stated that they had chronic diseases. 52.9% stated that they use public rather than private transport for travel (47.1%). In relation to the average daily travel time to university, 61% said it took less than one hour, 35.6% one to two hours, 3.4% more than two hours. As for degree programs: 19.4% economic area, 13.7% legal area, 35.4% humanities area, 15.8% engineering area, 15.7% health area.

### 2.4. Statistical Analysis

For the purposes of data analysis, three distinct groups of compilations received in the three time intervals (193 up to March 3, 185 from March 4 to March 8, 329 from March 9 to March 25) coinciding with the progressive containment measures implemented by the Italian Government were considered. The main analyses performed were: descriptive statistics to illustrate socio-demographic and health information, residence and the use of public transport for mobility; Pearson bivariate correlations for all main measures (Generalized Self-efficacy Scale, Locus of Control of Behavior, ITAPI-S, Self-Restraint Intentions, Risk Perception, Trust) significant at *p* < 0.005 and at *p* < 0.001, 2-tailed); Cronbach’s alpha as scale reliability coefficient; Anova one-way with post-hoc Tukey HSD and *p* < 0.05 to explore significant differences within the three time intervals considered, and Cohen’s f as a measure of effect size (0.1: small; 0.25: medium; 0.40: large) were used. Linear hierarchical regression to identify the predictors of self-efficacy and behavioural restraint intentions. Simple mediation analysis to test the function of trust on the effects of risk perception on self-restraint intentions. The mediation analysis was performed through the PROCESS macro version 3.3 (www.processmacro.org, accessed on 28 July 2019) [33]. The considered indexes were all subjected to an EFA and PCA exploratory verification.

## 3. Results

### 3.1. Descriptive Statistics

No significant influences resulted on the perception of risk depending on the size of the place of residence (cities, towns, small towns), gender, age, degree course. Participants worried about being infected in public transport (M = 4.18 SD = 1.09), shops or restaurants (M = 4.02 SD = 1.20) more than by visiting their family and friends (M = 3.16 SD = 1.46; *p* < 0.001). Individuals with chronic diseases obviously showed greater sensitivity, perceiving the risk of contagion as more dangerous (*p* < 0.05).

As for behavioral self-limitation intentions, the variables of gender, age, area of residence, and degree program did not show significance.

In contrast, those with chronic illnesses showed greater behavioral restraint intentions (M = 3.84 SD = 1.01; *p* < 0.05) than others (M = 3.47 SD = 1.11). Those who usually use public transportation showed lower self-restraint intention (M = 3.41 SD = 1.10) than those who usually use private transportation (M = 3.60 SD = 1.10; *p* < 0.05).

The comparability of the three groups was verified by testing for non-significant differences in their internal composition with respect to the variables of area of residence, degree course, gender, means of transportation and presence of chronic diseases.

Table 1, Table 2, Table 3, Table 4, Table 5 and Table 6 below report the comparison by chi-square test of the descriptive characteristics of the three groups. The corresponding dataset is available as Supplementary Material File S1.

All correlations between the scale variables used are presented in Table 7 below.

The table shows firstly the strong correlation between risk perception and social distancing (0.649 **) and vulnerability (0.135 **). Self-efficacy has strong positive correlations with dynamism (0.439 **), internal locus (0.413 **), conscientiousness (0.256 **), and negative correlations with external locus (−0.305 **), vulnerability (−0.290 **) and self-restraint intentions (−0.182 **). As far as the orientation of the locus is concerned, the internal locus strongly correlates with dynamism (0.385 **), while the external locus correlates with vulnerability (0.478 **). Trust correlates positively with both risk perception (0.270 **) and self-restraint intentions (0.262 **).

### 3.2. Change in Risk Perception

The risk perception indicator showed significant increasing variations in the three intervals, with a particular increase in the average for the third interval, coinciding with the restrictive measure of home quarantine for the entire population. A one-way Anova was computed by comparing the scores of subjects who were tested under the three different conditions: F(2,706) = 132.54 *p* < 0.001 M_1_ = 2.67 SD = 0.57; M_2_ = 2.92 SD = 0.62; M_3_ = 3.47 SD = 0.53. Tukey’s HSD post-hoc analysis demonstrated that all three averages show significant differences (Subset for alpha = 0.05). Overall effect size was large: f = 0.61.

### 3.3. Variation in Perceived Self-Efficacy

The measure of perceived self-efficacy showed a significant decrease corresponding to the third interval, coinciding with the restrictive quarantine measure. Anova one-way: F(2,76) = 22.605 *p* < 0.001 M_1_ = 3.82 SD = 0.58; M_2_ = 3.85 SD = 0.71; M_3_ = 3.50 SD = 0.74;. Tukey’s HSD showed that the average of the third range was significantly lower than the other two (Subset for alpha = 0.05). Overall effect size was medium: f = 0.26.

### 3.4. Change in the Attribution of Value to Social Responsibility

The assessment of the importance of individual protection measures as a duty to the community showed a significant increase in the progression of the ranges considered. Anova one-way: F(2,715) = 42.842 *p* < 0.001 M_1_ = 3.95 SD = 1.00; M_2_ = 4.26 SD = 0.91; M_3_ = 4.63 SD = 0.66. Tukey’s HSD demonstrated that all three averages show significant differences (Subset for alpha = 0.05). Overall effect size was medium: f = 0.34.

### 3.5. Change in Perceived Trust in the Behavior of Others

The comparison between the measures of confidence in the social responsibility of others showed a significant drop in the second interval, coinciding with the first measures of school and university closures and the spread of cases of contagion in the Lombardy and Veneto regions. In the first interval, coinciding with the increase in the level of risk with the communication of the initial cases in Northern Italy, confidence in the sense of collective responsibility was instead sufficient. Anova one-way: F(2,706) = 22.027 *p* < 0.001 M_1_ = 2.35 SD = 0.95; M_2_ = 1.87 SD = 0.91; M_3_ = 2.39 SD = 0.85. Tukey’s HSD showed that the average of the second range was significantly lower than the other two (Subset for alpha = 0.05). Overall effect size was medium: f = 0.25.

### 3.6. Change in Perceived Confidence in Health Authority Decisions

The comparison between the measures of confidence in the efficiency of health authorities in the management of the emergency showed a significant decrease in the second interval, coinciding with the first measures of school and university closures and the spread of cases of contagion in the Lombardy and Veneto regions, and a significant increase in the third interval. Anova one-way: F(2,706) = 30.849 *p* < 0.001 M_1_ = 2.94 SD = 1.04; M_2_ = 2.62 SD = 0.92; M_3_ = 3.33 SD = 1.02. Tukey’s HSD showed that the mean in the second interval was significantly lower than the other two, while it was significantly higher in the third interval (Subset for alpha = 0.05). Overall effect size was medium (f = 0.30).

### 3.7. Change in Perceived Confidence in Government Provisions

The comparison of the measures of confidence in the Government’s provisions in the management of the emergency showed a significant increase in the third interval, coinciding with the stricter provisions of containment and social isolation. Anova one-way: F(2,706) = 13.008 *p* < 0.001 M_1_ = 2.89 SD = 0.73; M_2_ = 2.98 SD = 0.81; M_3_ = 3.22 SD = 0.73. Tukey’s HSD showed that the average of the third interval was significantly higher than the other two, which were substantially homogeneous (Subset for alpha = 0.05). Overall effect size was small (f = 0.19). Table 8 below shows an overview of the significant variations in the scores of the variables in the three periods.

### 3.8. Variation of Self-Restraint Behaviours

Table 9 below illustrates how the intentions to limit the use of public transport, attendance at entertainment venues, purchasing essential goods, limiting attendance at work/university, attendance at medical practices showed a significantly increasing trend in the three intervals. As regards visiting friends and relatives and the need to stay at home as much as possible, there was no significant difference between the first and second periods. Therefore, as long as there was no requirement to stay in isolation at home, it was apparent that the participants intended to continue meeting friends even in open spaces.

### 3.9. Predictors of Perceived Self-Efficacy

The preliminary verifications of the regression assumptions excluded the presence of multivariate outliers. Mardia’s multivariate kurtosis index (75.944) was in fact below the critical value [*p* (*p* + 2) = 63]; therefore the relationship between the variables can be considered substantially linear. Low co-linearity was indicated by the low variance inflation factor (VIF) values < 2 and high tolerance values >0.60. For verification of the assumptions on the residuals, the average between the standardized and raw residuals was equal to 0; the Durbin–Watson test had a value of 2.17 and was therefore indicative of the absence of autocorrelation.

The hierarchical regression analysis identified in the dynamism trait of the person the most relevant explanatory weight (β = 0.307 and ΔR^2^ = 0.193) on the variation of self-efficacy levels, while for the vulnerability component a negative β (−0.193 and ΔR^2^ = 0.047) and a positive β for the defensiveness trait (β = 0.088 and ΔR^2^ = 0.006) emerged. Second, the internal locus of control was identified as an influential positive predictor (β = 0.241 and δr^2^ = 0.070) and the external locus of control as a negative predictor (β = −0.085 and ΔR^2^ = 0.005) of perceived self-efficacy. Finally, the risk perception proved to be a negative predictor of self-efficacy (β = −0.092 and ΔR^2^ = 0.008). The model has a total R^2^ = 0.329.

### 3.10. Predictors of Self-Restraint Intentions

The preliminary verifications of the regression assumptions excluded the presence of multivariate outliers. Mardia’s multivariate kurtosis index (22.63) was in fact below the critical value [*p* (*p* + 2) = 24]; hence the relationship between the variables can be considered substantially linear. Low co-linearity was indicated by the low VIF values () < 2 and high tolerance values >0.60. For verification of the assumptions on the residuals, the average between the standardized and raw residuals was equal to 0; the Durbin–Watson test had a value of 2.16 and was therefore indicative of the absence of autocorrelation.

The hierarchical regression analysis identified as significant predictors of self-restraint intentions the risk perception (β = 0.610 with ΔR^2^ = 0.421), self-efficacy (β = −0.154 and ΔR^2^ = 0.021), trust (in government, others and health authorities) (β = 0.093 and ΔR^2^ = 0.008), degree course (β = 0.069 with ΔR^2^ = 0.005) and age (β = 0.062 with ΔR^2^ = 0.004). R^2^ = 0.459.

### 3.11. Trust as Mediator of Risk Perception on Self-Restraint Intentions

**Figure 1:**
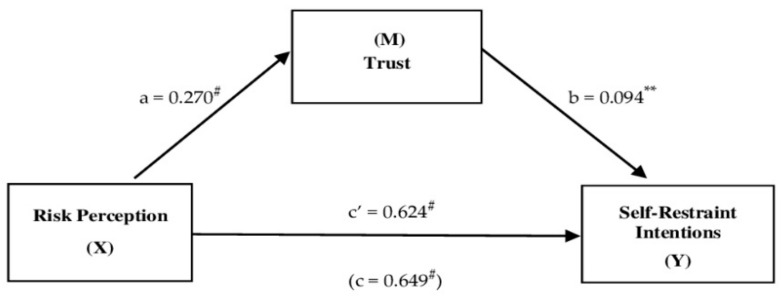
The mediating effect of trust in the relationship between risk perception and self-restraint intentions. Notes: **^#^** *p* < 0.0001; ** *p* < 0.001; all presented effects are standardized; a is effect of Risk Perceptions on Trust; b is effect of Trust on Self-Restraint Intentions; c’ is direct effect of Risk Perceptions on Self-Restraint Intentions, c is total effect of Risk Perceptions on Self-Restraint Intentions.

Considering the main beta coefficients associated with the predictors of the previous hierarchical regression model on the self-restraint intentions variable, the mediation of trust on the effect of risk perception was hypothesized. The other predictors (self-efficacy, age, and degree program), once entered as covariates in the model, were not significant. Therefore, results from a simple mediation analysis, as shown in Figure 1 below, indicated that risk perception was indirectly related to self-restraint intentions through its relationship with trust. firstly, as can be seen in Figure 1, risk perception had a positive effect on trust (a = 0.270, *p* = 0.000), and a higher reported trust was subsequently related to more self-restraint intentions (b = 0.094, *p* = 0.001). A 95% bias-corrected confidence interval based on 10,000 bootstrap samples indicated that the indirect effect (ab = 0.025) was entirely above zero (0.009 to 0.045). Moreover, higher levels of risk perception corresponded to higher self-restraint intentions even after taking into account risk perception’s indirect effect through trust (c’ = 0.624, *p* = 0.000).

## 4. Discussion

We aimed to investigate the change in risk perception, perceived self-efficacy, and the perceived trust in the behaviour of others, the decisions of health authorities and government provisions, and the variation of self-restraint behaviours during the spread of the Covid-19 pandemic among university students in Italy. Results confirmed that significant changes in the time progression occurred in the perception of risk, in the perception of individual self-efficacy, in the value attributed to social responsibility, in interpersonal trust and in trust in health authorities.

Perception of risk, in relation to our sample who resided in central-southern Italy, showed a progressive increase though the three-time intervals with a significant increase in the period coinciding with the quarantine imposed on the entire population.

These results are similar to those from a recent study in the United States that showed a rapid increase in risk perception for Covid-19 over a short time period, perhaps correlated to public health messages spread through the government and the media [34]. This showed us the need for greater public health efforts, mainly considering the most vulnerable communities.

The measure of self-efficacy showed a significant decrease in the period of confinement at home (lockdown). In the first and second time interval, the measure of self-efficacy remained substantially at medium-high levels and higher than those shown by the trend of risk perception. There was probably a low perception of the actual risk of contagion, in favour of a significant confidence in one’s ability to manage and cope with the situation, as well as to escape contagion since the spread of the virus was still mainly limited to areas in northern Italy. Starting from 9 March, the two trends presented an important variation that almost leads the average values of the two variables to coincide, i.e., a strong increase in risk perception corresponded to a sharp decrease in self-efficacy perceived by the subjects.

This result agrees with the findings of a study on avian influenza that showed that self-efficacy was inversely associated with risk perception [4] and with more recent studies on Covid-19 [35,36,37]. Considering Turkish adults, Yıldırım and Güler found that people that showed high self-efficacy and engagement in preventive behaviors had better mental health during the pandemic [35].

Another important aspect is the observation of the confidence variable in the provisions indicated by the authorities. In our study, the attribution of trust showed a significantly marked drop in the interval between 4 and 8 March 2020, when the first measures were issued, closing schools and universities, blocking participation in public and sports events, and restricting access to visiting health and prison facilities. The reaction to these first measures tended to be critical, perhaps due to the subjects’ difficulty in accepting the first behavioral limitations, considering them inadequate and slightly confusing.

In two preliminary studies on individual perceptions and awareness during the early stage of Covid-19 in the US and Finland, the trust and confidence in political authorities’ actions to prevent the outbreak was quite low [1,25] while more recent studies showed that the implementation of restrictive measures increased trust in the government, especially for individuals who didn’t experience the disease, directly or indirectly [26,27]. An interesting study conducted in Serbia during the first wave of the Covid-19 pandemic assessed the influence of conspiracy beliefs and political trust on adherence to COVID-19 preventive behaviour [28]. Conspiracy beliefs include hiding information and the belief that the virus is harmless. Here results indicated that holding more conspiracy beliefs was related to less adherence to containment-related behaviour, both directly and indirectly, via decreased political trust.

In our case, following the enactment of a more drastic set of measures, which also involved the productive and commercial sectors of the country, there was a rise in general confidence, as though a greater awareness of commitment and collective involvement in the challenge of facing the danger had emerged. Worthy of particular attention is the polarized trend between the expectations of social responsibility (for individuals called upon to implement individual protection and precautionary measures as an ethical duty towards the community) and the trust placed in the real behaviour of others. While on the one hand, ethical expectations were very high, on the other, trust in the behaviour of others was very low. Throughout all three periods considered, this gap remained almost constant.

A study on different countries that used cross-national index data from the World Value Survey showed that the level of social trust influences the speed of the transmission of Covid-19. In countries with a high level of social trust, the number of new infections tended to reach the first peak within a shorter time duration than in other countries [37].

Trust is associated with a greater compliance with policy measures [38]. Understanding the dynamics of risk perception and general trust is very important in order to predict the intention to implement precautionary behaviour in times of emergency.

In our study it is significant to note that before the restrictive home confinement measure of 8 March, there was no change in the intention to limit contact with friends and relatives or to stay at home as much as possible during the two previous periods. Although there was a massive and continuous communication campaign initiated by the authorities on the importance of preventing the spread of the virus through the practice of social distancing and a better hand hygiene, cases of infection and spread caused by meetings, attendance and gatherings, especially among young people, continued to be recorded. Only after the decree of 8 March and thereafter was there a significant increase in intentions of precautionary limitation in behaviour.

As demonstrated in previous studies about other pandemics and in a recent study on Covid-19, perceived personal risk influences the use of protective behaviours such as hand washing and social distancing; therefore, the higher perception of risk that we recorded in the third period of the lockdown was predictive for the engagement in protective behaviours [39,40].

The preliminary analysis of hierarchical regression carried out in our study on the self-efficacy variable identified six significant predictors: three traits, two related to the orientation of causal attribution, one related to the perception of risk, i.e., dynamism (positive predictor), vulnerability (negative predictor) and defensiveness (positive predictor); internal locus of control (positive predictor) and the external locus (negative predictor); risk perception (negative predictor).

This result is in line with the findings of research on the determinants of risk perception showing that individuals with internal locus of control and high self-efficacy should perceive risk higher than their counterpart [41]. We can therefore say that, as in the literature [12], when individuals are assessed with scores typical of the internal locus of control, they can better deal with emergency situations because they feel they are an active part in the management of the situation, moved by conscientiousness and empathy, and show a greater interpersonal and institutional trust; while individuals characterized by a main trait component of vulnerability and who are likely to feel less able to control and face difficult situations, tend to have a low level of self-efficacy, even if they try to protect themselves by assuming attitudes of defensive closure. On the whole, their attitude is fatalistic and not very conscientious; the sense of personal vulnerability is associated with a high perception of risk, in response to which they activate defensive instances associated with social closure, lack of interpersonal and institutional trust.

With regard to the self-restraint intentions, a second hierarchical regression was carried out on this variable, and the perception of risk, trust and self-efficacy showed main significance as positive predictors. therefore, it can be said that certainly those who have a greater perception of risk are also those who adapt better to the provisions indicated by the government and health authorities, implementing behaviours that limit daily activities both at the social and individual level. However, it is not only and primarily the fear of risk that drives people to engage in precautionary behaviour, but also the level of institutional and interpersonal trust. A recent systematic review on the consequences of trust on the Covid-19 pandemic has been proposed by Devine et al., showing how trust is associated with greater compliance with policy measures. At the same time, it also suggests that not only who delivers the measures but also the attitudes of those around the person mediate this relationship [27].

Our subsequent model of mediation demonstrated its adequacy with respect to the hypothesis, indicating that risk perception exerts a direct effect on the intention to implement precautionary behaviour and that this effect is greater if mediated by trust: as interpersonal and/or institutional trust increases, the impact of the risk perception on the precautionary behaviour assumed increases.

In our case, in the initial stages of the spread of the contagion in northern Italy, a low level of both institutional and interpersonal trust was recorded. In the second period, institutional confidence registered a further significant decline. This means that the influence of a component that could have helped to steer collective behaviour towards the responsible adoption of precautionary measures at an early and timely stage was lacking until 8 March. Instead, we had to wait for the massive increase in the perception of risk and danger resulting from the rapid spread of contagion to the other regions of central and southern Italy and the growing and threatening number of deceased patients in northern Italy. Therefore, also the social and institutional trust component should be considered as important protective resource in cases of collective emergency such as the current pandemic. Trust (in health/political authorities and in the behaviour of others) plays an influential role in the model, also in terms of prospective consideration. Therefore, the concluding results of the study suggest investing in the consolidation of trust in institutions and paying more attention to stimulating a sense of social cohesion, in order to increase collective responsiveness in the timely development of a unified and proactive attitude in the management of serious risk situations such as pandemic risk. Institutional and interpersonal trust are therefore essential factors/resources of protection and resilient community response.

### Limitations and Strengths

This study has some limitations that should be addressed. First of all, the main limitation is that the three groups of participants corresponding to the three intervals examined were composed of different groups of people. A more accurate measure of variation in the variables considered would have required that the same people be re-contacted in the second and third time intervals.

An extension of the study to other age groups should be considered in order to confirm the patterns identified, even though the findings of the literature about age differences in risk perception are controversial [38].

Moreover, our study didn’t take into consideration the socio-economic stratification, a variable that, according to previous studies, could lead to different subjective perceptions of risk depending on people’s socioeconomic status [39].

A final limitation of the study is the fact that the participants come exclusively from the central-southern part of Italy; the results and models should therefore be verified with a further extension of the sample to other areas of the country.

The strength and innovation of our study is related to the characteristics of the sample and the study’s contribution to a better understanding of human behaviour during the pandemic. The sample of 707 young university students investigated can be considered representative of the student community that is the target of this research. The university student community is a very interesting group to study in relation to Covid-19, as its great mobility, its socialization habits, its social network and its difficulties with social distance can have a significant impact on the spread of the virus [37].

In our investigation about the perception of risk, the analysis of the role of social trust in combination with trust in health and government authorities and self-efficacy can also be considered a very original aspect since many of the previous studies analyze the relationship with only one or two variables at the same time.

The final mediation model is intended to be our original contribution to a better understanding of the factors that can positively affect the activation and maintenance of precautionary behaviours, crucial in the management of a pandemic emergency.

The validity of the model hypothesized and tested in this study with undergraduate students will need to be further tested with larger samples which also have different characteristics. Further research may possibly also test for the existence of correlates and/or antecedents of trust the could directly or indirectly via trust predict self-restraint behaviors.

## 5. Conclusions

The present study showed that the time interval and the corresponding measures taken by the government in order to prevent and contain the pandemic influenced the perception of risk, the perception of individual self-efficacy, the value attributed to social responsibility, interpersonal trust and trust in health authorities.

Especially in the first phase, youths’ measures of social distancing were significantly mild, showing a reluctance to forgo personal contact with friends and relatives.

With the extension and increase of the restrictive measures, their perception of risk changed, as well as their self-efficacy, trust in others and health and government authorities. Students with a higher perception of risk put in place higher self-restrain and preventive behaviours when they trusted others and health and government authorities.

Considering the great influence that social trust appeared to have on students’ behaviours and beliefs, the sense of community and cooperation among people to achieve common goals should be promoted and supported.

As reported by Fahlquist [24], decreasing the spread of the pandemic also requires that individuals take personal responsibility: each individual has a responsibility to understand that one’s own decisions necessarily have an impact on other people. For people to be willing to take responsibility to develop the habits necessary for managing a pandemic, they need to trust their government, but trust is relational and requires that the trusted party be worthy of trust.

Governments must communicate clearly how they balance conflicts between collective health and individual rights and values and what the chosen strategy entails in terms of collective and individual responsibility. Individuals need to be involved in determining which decisions are made and how decisions are made. Governments must therefore facilitate the development of trust and solidarity with and among citizens [24].

Since the target of our study was focused on young university students, the indications of strengthening the sense of social responsibility and institutional trust must necessarily be accompanied by the need to implement language and actions for involvement which are truly adapted to the characteristics and needs that characterize this age group.

The conclusing results of the study suggest stimulating in young people more trust in institutions, a higher sense of social cohesion and responsibility and, as also recommended by Alessandri et al. [42], investing in civic education programs or, more generally, in all those interventions that may empower civic engagement, in order to increase their collective responsiveness in the management of serious risk situations such as pandemic risk.

## Figures and Tables

**Table 1:** Time interval in relation to participants’ area of residence.

Area of Residence			City	Town	Small Town	Total
**Interval**	from 25 Febrary to 3 March	Count	14	78	101	193
		% within Intervals	7.3%	40.4%	52.3%	100.0%
		% within Residence	31.8%	31.3%	24.4%	27.3%
	from 4 March to 8 March	Count	11	58	116	185
		% within Intervals	5.9%	31.4%	62.7%	100.0%
		% within Residence	25.0%	23.3%	28.0%	26.2%
	from 9 March to 25 March	Count	19	113	197	329
		% within Intervals	5.8%	34.3%	59.9%	100.0%
		% within Residence	43.2%	45.4%	47.6%	46.5%
Total		Count	44	249	414	707
		% within Intervals	6.2%	35.2%	58.6%	100.0%
		% within Residence	100.0%	100.0%	100.0%	100.0%

Chi-Square Test: 4.713; df = 4; Asymp. Sig. (2-sided) = 0.318.

**Table 2:** Time interval in relation to participants’ degree course.

Degree Course			Economics	Law	Humanities	Engineering	Health	Total
**Interval**	from 25 Febrary to 3 March	Count	40	31	57	31	34	193
		% within Intervals	20.7%	16.1%	29.5%	16.1%	17.6%	100.0%
		% within Degree	29.2%	32.0%	22.8%	27.7%	30.6%	27.3%
	from 4 March to 8 March	Count	42	26	74	23	20	185
		% within Intervals	22.7%	14.1%	40.0%	12.4%	10.8%	100.0%
		% within Degree	30.7%	26.8%	29.6%	20.5%	18.0%	26.2%
	from 9 March to 25 March	Count	55	40	119	58	57	329
		% within Intervals	16.7%	12.2%	36.2%	17.6%	17.3%	100.0%
		% within Degree	40.1%	41.2%	47.6%	51.8%	51.4%	46.5%
Total		Count	137	97	250	112	111	707
		% within Intervals	19.4%	13.7%	35.4%	15.8%	15.7%	100.0%
		% within Degree	100.0%	100.0%	100.0%	100.0%	100.0%	100.0%

Chi-Square Test: 12.699; df = 8; Asymp. Sig. (2-sided) = 0.123.

**Table 3:** Time interval in relation to participants’ gender.

Gender			Male	Female	Total
**Interval**	from 25 Febrary to 3 March	Count	76	117	193
		% within Intervals	39.4%	60.6%	100.0%
		% within Gender	25.8%	28.4%	27.3%
	from 4 March to 8 March	Count	75	110	185
		% within Intervals	40.5%	59.5%	100.0%
		% within Gender	25.4%	26.7%	26.2%
	from 9 March to 25 March	Count	144	185	329
		% within Intervals	43.8%	56.2%	100.0%
		% within Gender	48.8%	44.9%	46.5%
Total		Count	295	412	707
		% within Intervals	41.7%	58.3%	100.0%
		% within Gender	100.0%	100.0%	100.0%

Chi-Square Test: 1.109; df = 2; Asymp. Sig. (2-sided) = 0.574.

**Table 4:** Time interval in relation to participants’ age group.

Age Group			18–21	22–24	25–36	Total
**Interval**	from 25 Febrary to 3 March	Count	69	57	67	193
		% within Intervals	35.8%	29.5%	34.7%	100.0%
		% within Age Group	24.6%	32.6%	26.7%	27.3%
	from 4 March to 8 March	Count	79	44	62	185
		% within Intervals	42.7%	23.8%	33.5%	100.0%
		% within Age Group	28.1%	25.1%	24.7%	26.2%
	from 9 March to 25 March	Count	133	74	122	329
		% within Intervals	40.4%	22.5%	37.1%	100.0%
		% within Age Group	47.3%	42.3%	48.6%	46.5%
Total		Count	281	175	251	707
		% within Intervals	39.7%	24.8%	35.5%	100.0%
		% within Age Group	100.0%	100.0%	100.0%	100.0%

Chi-Square Test: 4.233; df = 4; Asymp. Sig. (2-sided) = 0.377.

**Table 5:** Time interval in relation to participants’ transport means.

Transport Means			Public Transport	Private Vehicles	Total
**Interval**	from 25 Febrary to 3 March	Count	111	82	193
		% within Intervals	57.5%	42.5%	100.0%
		% within Transport means	29.7%	24.6%	27.3%
	from 4 March to 8 March	Count	96	89	185
		% within Intervals	51.9%	48.1%	100.0%
		% within Transport means	25.7%	26.7%	26.2%
	from 9 March to 25 March	Count	167	162	329
		% within Intervals	50.8%	49.2%	100.0%
		% within Transport means	44.7%	48.6%	46.5%
Total		Count	374	333	707
		% within Intervals	52.9%	47.1%	100.0%
		% within Transport means	100.0%	100.0%	100.0%

Chi-Square Test: 2.329; df = 2; Asymp. Sig. (2-sided) = 0.312.

**Table 6:** Time interval in relation to participants’ chronic deseases.

Chronic Deseases			Yes	No	Total
**Interval**	from 25 Febrary to 3 March	Count	10	183	193
		% within Intervals	5.2%	94.8%	100.0%
		% within Chronic deseases	20.0%	27.9%	27.3%
	from 4 March to 8 March	Count	9	176	185
		% within Intervals	4.9%	95.1%	100.0%
		% within Chronic deseases	18.0%	26.8%	26.2%
	from 9 March to 25 March	Count	31	298	329
		% within Intervals	9.4%	90.6%	100.0%
		% within Chronic deseases	62.0%	45.4%	46.5%
Total		Count	50	657	707
		% within Intervals	7.1%	92.9%	100.0%
		% within Chronic deseases	100.0%	100.0%	100.0%

Chi-Square Test: 5.187; df = 2; Asymp. Sig. (2-sided) = 0.075.

**Table 7:** Pearson’s bivariate correlations.

Variables	RPE	GSE	ILC	ELC	VUL	EMP	CON	IMA	DEF	DYN	INT	TRU	SRI
RPE	1												
GSE	−0.057	1											
ILC	0.124 **	0.413 **	1										
ELC	0.042	−0.305 **	−0.306 **	1									
VUL	0.135 **	−0.290 **	−0.139 **	0.478 **	1								
EMP	0.097 **	0.162 **	0.230 **	−0.055	0.079 *	1							
CON	0.101 **	0.256 **	0.296 **	−0.132 **	−0.115 **	0.296 **	1						
IMM	0.044	0.126 **	0.164 **	0.080 *	0.270 **	0.266 **	0.111 **	1					
DEF	0.088 *	0.087 *	0.142 **	0.136 **	0.247 **	0.097 **	0.186 **	0.185 **	1				
DYN	0.087 *	0.439 **	0.385 **	−0.201 **	−0.107 **	0.205 **	0.344 **	0.310 **	0.106 **	1			
INT	0.039	0.036	0.099 **	0.096 *	0.282 **	0.038	0.059	0.160 **	0.167 **	0.031	1		
TRU	0.270 **	−0.013	0.072	−0.065	−0.079 *	0.160 **	0.169 **	−0.020	−0.059	0.039	−0.153 **	1	
SRI	0.649 **	−0.182 **	−0.009	0.037	0.094 *	0.086 *	0.017	0.047	−0.007	−0.025	0.075 *	0.262 **	1
SKE (SE)	0.013 (0.092)	−0.485 (0.092)	−0.363 (0.092)	0.242 (0.092)	0.017 (0.092)	−0.553 (0.092)	−0.332 (0.092)	−0.341 (0.092)	0.248 (0.092)	−0.231 (0.092)	−0.115 (0.092)	−0.107 (0.092)	−0.375 (0.092)
KUR (SE)	−0.356 (0.184)	0.185 (0.184)	0.263 (0.184)	0.046 (0.184)	−0.395 (0.184)	0.366 (0.184)	0.043 (0.184)	−0.203 (0.184)	−0.200 (0.184)	0.082 (0.184)	−0.481 (0.184)	−0.207 (0.184)	−0.907 (0.184)
M (SD)	3.11 (0.666)	3.68 (0.687)	4.58 (0.650)	2.68 (0.760)	2.72 (0.651)	3.25 (0.482)	2.98 (0.564)	3.09 (0.581)	3.09 (0.466)	2.99 (0.527)	2.80 (0.612)	2.92 (0.651)	3.50 (1.10)
alpha	0.704	0.852	0.730	0.715	0.723	0.694	0.703	0.737	0.687	0.701	0.714	0.717	0.923

Note: RPE = Risk Perception; GSE = General Self-Efficacy; ILC = Internal Locus of Control; ELC = External Locus of Control; VUL = Vulnerability; EM = Empathy; CON = Conscientiousness; IM = Imagination; DEF = Defensiveness; DYN = Dynamism; IN = Introversion; TRU = Trust; SRI = Self-Restraint Intentions; SKE = Skewness; KUR = Kurtosis; SE = Standard Error; M = Mean; SD = Standard Deviation; alpha = Cronbach’s alpha. *n*= 707, ** Correlation is significant at the 0.01 level (2-tailed). * Correlation is significant at the 0.05 level (2-tailed).

**Table 8:** Variations in average scores in the three intervals.

Variables	Range	F	M_1_ (SD)	M_2_ (SD)	M_3_ (SD)	*p*	ES (*f*)	ES Level
Risk Perception	1–5	132.538	2.67 * (0.57)	2.92 * (0.62)	3.47 * (0.53)	<0.001	0.61	large
Perceived Self-Efficacy	1–5	22.605	3.82 (0.58)	3.85 (0.71)	3.50 * (0.74)	<0.001	0.26	medium
Value to social responsibility	1–5	41.842	3.95 * (1.00)	4.26 * (0.91)	4.63 * (0.66)	<0.001	0.34	medium
Confidence in other people’s behavior	1–5	22.027	2.35 (0.95)	1.87 * (0.91)	2.39 (0.85)	<0.001	0.25	medium
Trust in health authorities	1–5	30.849	2.94 (1.04)	2.62 * (0.92)	3.33 (1.02)	<0.001	0.30	medium
Confidence in government regulations	1–5	13.008	2.89 (0.73)	2.98 (0.81)	3.22 * (0.73)	<0.001	0.19	small

Note: M_1_ = Interval 25 February to 3 March; M_2_ = Interval 4 March to 8 March; M_3_ = Interval 9 March to 25 March; N = 707; N_1_ = 193; N_2_ = 185; N_3_ = 329; SD = Standard Deviation; * = *p* < 0.05; EF = Effect Size; *f* = Cohen’s f.

**Table 9:** Variation of self-restraint intentions in the three intervals.

Variables	Range	F	M_1_ (SD)	M_2_ (SD)	M_3_ (SD)	*p*	ES (*f*)	ES Level
Avoid Public Transport	1–5	130.967	3.51 * (1.20)	3.81 * (1.13)	4.77 * (0.55)	<0.001	0.55	large
Avoid Public Venues	1–5	188.258	3.26 * (1.24)	3.46 * (1.20)	4.78 * (0.58)	<0.001	0.66	large
Limit Purchases	1–5	178.160	2.56 * (1.24)	2.88 * (1.23)	4.29 * (0.96)	<0.001	0.67	large
Abstention from Work/University	1–5	122.273	1.93 * (1.08)	2.22 * (1.20)	3.52 * (1.34)	<0.001	0.59	large
Avoid Direct Contacts	1–5	288.782	2.18 (1.15)	2.32 (1.13)	4.21 * (1.01)	<0.001	0.88	large
Avoid Medical Practices	1–5	115.782	2.67 * (1.23)	3.97 * (1.30)	4.12 * (1.00)	<0.001	0.54	large
Stay at Home	1–5	213.202	2.68 (1.30)	2.76 (1.27)	4.46 * (0.89)	<0.001	0.74	large

Note: M_1_ = Interval 25 February to 3 March; M_2_ = Interval 4 March to 8 March; M_3_ = Interval 9 March to 25 March; N = 707; N_1_ = 193; N_2_ = 185; N_3_ = 329; SD = Standard Deviation; * = *p* < 0.05; EF = Effect Size; *f* = Cohen’s f.

## Data Availability

The dataset used for this study is available as supplementary material.

## Supplementary Materials

The following is available online at https://www.mdpi.com/1660-4601/18/7/3427/s1, File S1: Perception_of_Risk_Covid.sav (dataset).
